# PAKs supplement improves immune status and body composition but not muscle strength in resistance trained individuals

**DOI:** 10.1186/1550-2783-7-36

**Published:** 2010-11-08

**Authors:** Frederigo G Romero, Fabio S Lira, Fernando A Marques, Paulo C Muzy, Rodolfo AN Peres, Érico C Caperuto

**Affiliations:** 1 Department of Physiology, Institute of Biomedical Sciences, University of São Paulo, Brazil; 2 Department of Physiology, Division of Nutrition Physiology, Federal University of São Paulo, Brazil; 3 Department of Biodynamic, Mackenzie Presbiterian University, São Paulo, Brazil; 4 Institute of Science in Nutrition and Performance, São Paulo, Brazil

## Abstract

Mixed formula supplements are very popular among recreational and professional weightlifters. They are usually known as PAKs and they are supposed to have a synergistic effect of their different nutrients. The purpose of this study was to determine the effects of chronic (4 weeks) PAKS supplementation in combination with strength training on body composition, immune status and performance measures in recreationally trained individuals with or without PAKs supplementation. Methods: Twelve male subjects (Placebo n = 6 and PAKs supplement n = 6) were recruited for this study. The body composition, one maximum strength repetition tests and immune status were assessed before and after 4 week supplementation. Our data showed that, 4 week PAK supplementation associated with strength exercise not was effective in change strength than compared with placebo group. However, we observed that, PAK supplement was able to improve immune status and reduced body composition when compared with placebo group. These results indicate that, a mixed formula supplement is able to improve immune status and body composition but not maximum strength in recreational strength trained subjects in a 4 weeks period.

## Introduction

Many dietary supplements are made commercially available in what is commonly referred to as PAKS. PAKS typically include several different pills and/or Tablets packaged in the same envelope to be ingested together.

The original idea on these products, according to manufacturers [1] was to facilitate consumers the lifestyle, supplying all the substances and nutrients needed for one training session or any specific situation in a single dose, instead of taking it from several bottles or products with varying dosages.

From the nutritional standpoint, a very important feature of these PAKS is that they deliver several components in a unique dose. Alone, these compounds are already known and have their nutritional properties established, however, when combined, they promote maximum performance on natural physiologic processes [2], as some compounds may serve as an energy source [3], as coenzymes in pathways that are specially important for exercise [2,3] and as ergogenic aids that might help to improve exercise performance [4]. When these properties are added, a combined effect is created resulting in higher performance and other benefits to the individuals.

Sport supplement use among active people, especially those interested in gaining muscle mass, is very popular for those seeking better and faster results [5]. Supplement manufacturers often bring innovating compounds or new combinations of known substances, in order to meet market demands. Most of the times, the market need for innovation and production speed overcome scientific evidence regarding these innovations. Thus, little is known about the chronic effects of these new products.

This study evaluated the effects of a mixed formula supplement on performance, body composition and immune status of recreational weightlifters.

## Materials and methods

### Subjects

Twelve (n = 12) healthy and non-smoking males participated in this study. The subjects were divided in two groups, a Placebo (n = 6) [age 28.6 (6.9) years, height 174.0 (0.04) cm, weight 75.6 (10.2) kg] and PAKS (n = 6) [age 29.8 (5.7) years, height 177.0 (0.06) cm, weight 74.7 (4.4) kg]. The physical characteristics of both groups are described in Table 1. The benefits and risks of this study were explained to each participant before written consent was obtained. The study procedures were previously approved by the Ethics Committee of the Mackenzie Presbiterian University, São Paulo, Brazil. Placebo samples were specially produced by the manufacturer as requested by the researchers.

**Table 1:** Physical Characteristics

	Placebo Group	PAK Group
**Height (cm)**	174.00 ± 0.04	177.00 ± 0.06

**Weight (Kg)**	75.6 ± 10.2	74.7 ± 4.4

**Age (years)**	28.6 ± 6.9	29.8 ± 5.7

### Body composition and Strength training

Height, weight and body mass index were measured and body composition was estimated via seven-site skinfold as described by Jackson and Pollock [6].

**Figure 1:**
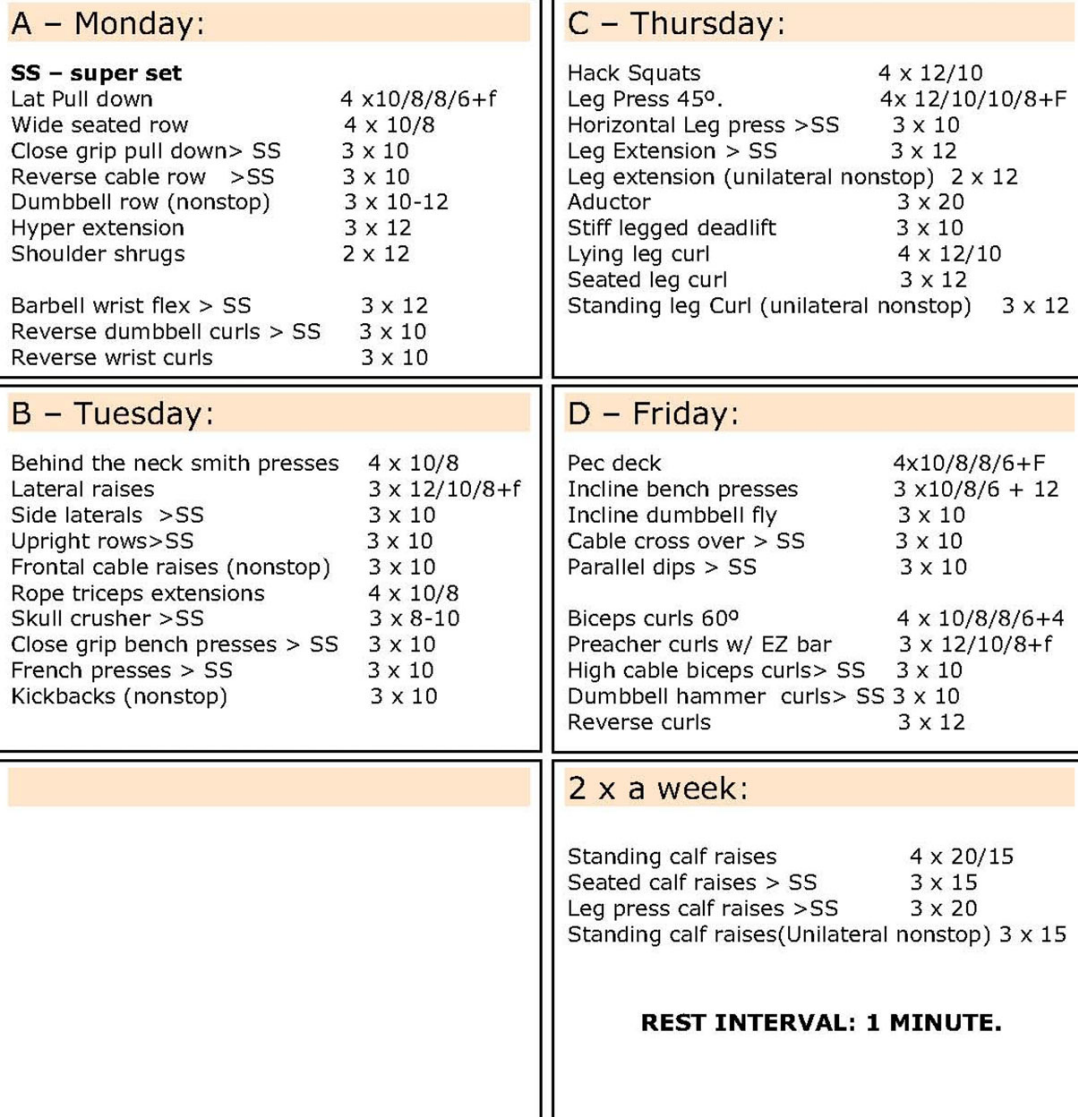
Training Routines

Strength training was composed of 4 different training routines that were performed each week. The training routines consisted of 4 sets of 10 or more repetitions at 80% one repetition maximal (1RM) with short rest intervals between sets (<60s). Specific exercise routines can be seen in Figure 1. One-repetition maximum (1RM) loads were determined prior to the initiation of the supplementation and after 4 weeks of training.

We evaluated performance in two exercises: bench press and lat pull down exercise with the One-repetition maximum test (1RM) as described by Brown and Weir [7].

### Dietary program

Energy intake was set at the levels recommended by the dietary reference intake for subjects with moderate levels of physical activity of the same age and gender following a balanced diet [8]. All subjects received individual nutritional consultation during the study; diets of all participants were balanced considering individual differences. Use of other supplements, other than the goal of this study and whey protein as prescribed by the nutritionist was not advisable, being considered as an exclusion factor. Subjects were oriented to ingest one PAK 30 minutes before the training session and every morning of non-training days.

### PAKs supplements composition

The studied supplement was a mixed formula that consisted of 11 elements in the form of tablets, capsules and pills. Their composition is shown in Table 2.

**Table 2:** PAK composition (one sachet)

	Amount in one sachet	Composition
**Big oval tablet**	1	2.3 g of protein

**Blue and black capsule**	1	64 mg of calcium, 22 mg of magnesium, 1.75 mf og zinc, 4 mg of niacin, 60 mcg of folic acid and 0.3 mg of B2 vitamin.

**Purple oval tablets**	2	22.5 mg of C vitamin.

**Small rounded pill**	1	5 mg of E vitamin

**Green and red capsule**	2	540 mg of guarana (22 mg of caffeine)

**Blue and white capsules**	2	316 mg of Isoleucin, 440 mg of Leucine, 316 mg of Valine and 0,3 mg of B6 vitamin

**White capsule**	1	200 mg of calcium

**Oval tablet**	1	500 mg of wheat protein

### Immune system evaluation

An upper respiratory tract infections questionnaire, adapted from Bassit and colleagues [9], as a report of the participant immune status. It's known that high intensity physical activity promotes light to moderate immune suppression [10], affecting the subject health and performance. The questionnaire is shown in Table 3 and consists of a list of symptoms or infections that may be marked by the subjects during the period of the study.

**Table 3:** Upper respiratory tract infections evaluation questionnaire

Symptoms Days	1	2	3	4	5	6	7	8	9	10	11	12	13	14	15	16	17	18	19	20	21
																					

Fever (°C)																					

Persistent muscle soreness (>than 8 h)																					

Pain in the next exercise session																					

Throat soreness																					

Throat mucus																					

Itchy or burning throat																					

Cough																					

Sneeze																					

Headache																					

Running nose																					

Cold																					

Flu																					

Herpes																					

Ulcers in the mouth																					

Conjunctivitis																					

Otitis																					

Mycosis																					

Candidiasis																					

Tendinitis																					

Articular pain																					

Sudden mood changes																					

Insomnia																					

Weakness																					

Anorexia																					

## Results

### Body composition results

Body composition and 1RM strength test are shown in Table 4.

**Table 4:** Results

Placebo Group		PAK Group	
**Body Fat Composition (% of body fat)**		**Body Fat Composition (% of body fat)**	

**Pre**	**Pos**	**Pre**	**Pos**

16.49 ± 1.52 (6)	16.67 ± 1.52 (6)	22.19 ± 0.55 (6)	20.13 ± 0.78* (6)

**1 MR Supine (Kg)**		**1 MR Supine (Kg)**	

**Pre**	**Pos**	**Pre**	**Pos**

98.00 ± 4.35 (6)	100.83 ± 3.97 (6)	91.00 ± 14.10 (6)	93.00 ± 13.38 (6)

**1 MR Pulley (Kg)**		**1 MR Pulley (Kg)**	

**Pre**	**Pos**	**Pre**	**Pos**

103.67 ± 1.33 (6)	106.67 ± 1.67 (6)	87.17 ± 12.54 (6)	95.83 ± 11.43 (6)

* p < 0,05 compared to Pre.

The placebo group didn't show any changes in body composition (before: 16.49 ± 1.52 and after: 16.67 ± 1.52), PAK group however, showed a significant decrease in body fat (before: 22.19 ± 0.55 and after: 20.13 ± 0.78).

For the one repetition maximum strength test, there were no significant changes between the groups. Supine values were 98.00 ± 4.35 kg before and 100.83 ± 3.97 kg after for the Placebo group and 91.0 ± 14.10 kg before and 92.00 ± 13.38 kg after for the PAK group. The same happened to the pulley exercise 1 MR, where values were 103.67 ± 1.33 kg before and 106.67 ± 1.67 kg after for the Placebo group, and 87.17 ± 12.54 kg before and 95.83 ± 11.43 kg after for the PAK group.

**Figure 2:**
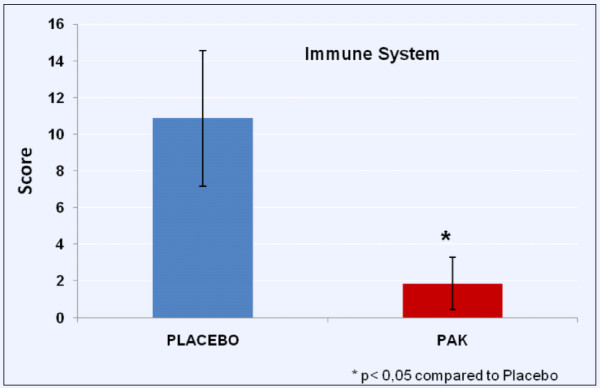
Immune System Status

Data for immune system status is shown in Figure 2.

Immune system activity was evaluated by the number of marks made in the questionnaire. Each mark meant a symptom or infection observed by the subject, therefore, the lower number of marks meant better immune system function. The placebo group showed higher marks (10.86 ± 3.69) than PAK group (1.86 ± 1.42) demonstrating maintenance of immune function.

## Discussion

Nutrition and training are key elements to change body composition, improve strength and modulate immune function [2,3]. Significant changes usually take time to occur and are generally associated to training and diet adherence. In the present study, it was observed that, improvement of immune status and reduced body fat composition in the subjects PAKs supplementation, with no significant effect on strength as measured by the 1RM bench press and lat pull down exercise.

Sport supplements are important tools to improve performance. Among them, there are nutritional aids that help to maintain health, also specially formulated nutrients and formulas that are widely used by athletes and sports enthusiasts. These supplements can decrease the time needed to improve muscle hypertrophy and body composition and maintain the immune status of people involved in high intensity exercise.

Immune system status depends on nutrition and general health but is also affected by high intensity exercises as described by Nieman [11] and Mackinnon [12]. These authors describe the benign influence of moderate intensity exercise on immune status and the negative influence caused by high intensity exercise or training. Although subjects submitted to stress, physical or emotional, or both, are more prone to infections, these effects can be mitigated by appropriate nutrition and rest. This immunosupression can be seen immediately after a high intensity exercise as well as during the entire training period. In the present study, it was shown that, short-term PAKs supplementation was able improves immune status in the subjects that participated in a high intensity strength exercise program. This may be an excellent strategy for the reduction of risk symptoms associated with the immunosupression situation. Multi-vitamins and mineral supplements are very useful to keep the immune system working properly [13], active people engaged in high intensity training or individuals who restrict energy intake, consume unbalanced diets (like those that promote extreme caloric restriction) may need supplements [14].

Still, we observed a reduction in body fat composition with subjects that utilized the PAKs supplementation after 4 weeks. Smith et al [15] showed a decrease of body fat and increase in muscle mass in a short interval (3 weeks) with a supplement that had caffeine, creatine and amino acids. Body composition changes, however, can be seen in hours or days, depending mainly on the magnitude of caloric restriction or training intensity. Ormsbee et al. [16] showed increased energy expenditure and fat oxidation immediately after a resistance exercise session, Gibala and McGee [17], showed changes in 2 weeks of high intensity exercise. Caffeine is a popular ergogenic aid with well described properties in the literature [4,18]. It's also known, that caffeine can change body composition, once it improves fat oxidation decreasing the body's fat mass [19]. Caffeine can be considered an ergogenic aid regarding fat oxidation from doses as low as 5 mg/kg [20].

On the other hand, we not found changes in the strength test after 4 weeks PAKs supplementation. Muscle hypertrophy usually is noted with up to 12 weeks of training [21], although a measureable strength improvement (due to factors other than muscle hipertrophy) can happen in as little as 2 to 4 weeks [22].

In conclusion, the use of the mixed formula supplement analyzed for 4 weeks was able to change body fat composition and maintain the immune system function but did not promote changes in strength in the recreational weightlifters that participated in this study. It's probable that a stronger nutrient combination may be able to show significant results in all the variables evaluated in this study.

## Competing interests

The authors declare that they have no competing interests.

## Authors' contributions

FAM developed the training routines and RANP organized the diets. PCM helped to develop and adapt the immune system evaluation and FGR, FSL and ECC conducted the research, collected and tabulated data. All authors discussed results and helped to write the discussion and conclusion. All authors read and approved the final manuscript.
