# Correction to: PAKs supplement improves immune status and body composition but not muscle strength in resistance trained individuals

**DOI:** 10.1186/s12970-018-0261-8

**Published:** 2018-11-27

**Authors:** Frederico G. Romero, Fabio S. Lira, Fernando A. Marques, Paulo C. Muzy, Rodolfo A. N. Peres, Érico C. Caperuto

**Affiliations:** 1 Department of Physiology, Institute of Biomedical Sciences, University of São Paulo, São Paulo, Brazil; 2 Institute of Science in Nutrition and Performance, São Paulo, Brazil; 3 Department of Physiology, Division of Nutrition Physiology, Federal University of São Paulo, São Paulo, Brazil; 4 Department of Biodynamic, Mackenzie Presbiterian University, São Paulo, Brazil

## Correction

The original article [1] contains an error whereby the author, Frederico G Romero’s name is displayed incorrectly; the correct spelling is instead displayed in this Correction article.
